# African Trypanosomosis Obliterates DTPa Vaccine-Induced Functional Memory So That Post-Treatment *Bordetella pertussis* Challenge Fails to Trigger a Protective Recall Response

**DOI:** 10.3390/vaccines9060603

**Published:** 2021-06-04

**Authors:** Magdalena Radwanska, Hang Thi Thu Nguyen, Stefan Magez

**Affiliations:** 1 Department of Biomedical Molecular Biology, Ghent University, Technologiepark Zwijnaarde 71, 9000 Ghent, Belgium; 2 Laboratory for Biomedical Research, Department of Molecular Biotechnology, Environment Technology and Food Technology, Ghent University Global Campus, Songdomunhwa-Ro 119-5, Yeonsu-Gu, Incheon 406-840, Korea; hang.nguyenthithu@ugent.be (H.T.T.N.); Stefan.Magez@vub.ac.be (S.M.); 3 Department of Biochemistry and Microbiology, Ghent University, Ledeganckstraat 35, 9000 Ghent, Belgium; 4 Laboratory of Cellular and Molecular Immunology, Department of Bioengineering Sciences, Vrije Universiteit Brussel, Pleinlaan 2, 1050 Brussels, Belgium

**Keywords:** trypanosomosis, treatment, DTPa, *Bordetella pertussis*

## Abstract

Salivarian trypanosomes are extracellular parasites causing anthroponotic and zoonotic infections. Anti-parasite vaccination is considered the only sustainable method for global trypanosomosis control. Unfortunately, no single field applicable vaccine solution has been successful so far. The active destruction of the host’s adaptive immune system by trypanosomes is believed to contribute to this problem. Here, we show that *Trypanosome brucei brucei* infection results in the lasting obliteration of immunological memory, including vaccine-induced memory against non-related pathogens. Using the well-established DTPa vaccine model in combination with a *T. b. brucei* infection and a diminazene diaceturate anti-parasite treatment scheme, our results demonstrate that while the latter ensured full recovery from the *T. b. brucei* infection, it failed to restore an efficacious anti-*B. pertussis* vaccine recall response. The DTPa vaccine failure coincided with a shift in the IgG1/IgG2a anti-*B. pertussis* antibody ratio in favor of IgG2a, and a striking impact on all of the spleen immune cell populations. Interestingly, an increased plasma IFNγ level in DTPa-vaccinated trypanosome-infected mice coincided with a temporary antibody-independent improvement in early-stage trypanosomosis control. In conclusion, our results are the first to show that trypanosome-inflicted immune damage is not restored by successful anti-parasite treatment.

## 1. Introduction

Salivarian trypanosomes are extracellular flagellated single-cell eukaryotic parasites that are known to colonize the mammalian bloodstream and lymphatics as well as the brain. More recently, fat tissue was identified as a major parasite survival location [1]. *Trypanosoma brucei rhodesiense* and *T. b. gambiense* are the agents of sleeping sickness, i.e., human African trypanosomosis (HAT) [2]. Animal trypanosomes (AT) are not infective to humans, as they are susceptible to lysis by the human serum protein APOL1 [3,4,5]. The species belonging to this category are *T. b. brucei* and the closely related *T. evansi*, *T. congolense*, *T. vivax* and *T. simiae* [6]. The ‘AT’ classification of *T. evansi* has, however, been called into question, as this parasite has been reported as the causative agent of several cases of atypical human trypanosomosis (aHT) in Asia [7,8,9]. *T. evansi* is also the most widely distributed pathogenic trypanosome, resulting from the fact that it can be mechanically transmitted by a range of biting flies and other blood-consuming vectors such as vampire bats. Hence, infections have been reported on all continents, including Europe [6]. Mechanical transmission also occurs in the case of *T. vivax* [10], while *T. congolense* is spread both through mechanical transmission [11] and the tsetse vector. Tsetses also transmit *T. b brucei* animal trypanosomosis and *T. b. gambiense*/*rhodesiense* sleeping sickness [12,13]. In trypanosomosis, the host anti-parasite response largely relies on the capacity to generate anti-trypanosome antibodies. The success of colonizing a broad range of hosts, including humans, infers however that trypanosomes acquired adaptation mechanisms allowing them to circumvent multiple immune killing mechanisms. These mechanisms have been best studied for *T. brucei*, with the cloned *T. b. brucei* AnTat 1.1 parasite serving as one of the most widely implemented in vivo and in vitro laboratory models [14,15]. The assessment of the host–parasite interaction of *T. brucei* at a molecular level, already accomplished more than 40 years ago, revealed that these parasites use antigenic variation in the variant surface glycoprotein (VSG) coat as a first line of defense against antibody-mediated killing [16,17,18,19,20]. This involves chromosomal recombination, expression site activation/silencing and access to an extensive reservoir of over 1000 genomic VSG genes and pseudogenes, allowing trypanosomes to ‘eternally’ outrun the mammalian adaptive immune system [21]. Interestingly, the elaborate VSG switching is not the only mechanism involved in parasite evasion of the host immune system, as there are severe limitations to the efficacy of antigenic variation. Indeed, as the trypanosome (i) does not have access to an unlimited reservoir of immunologically distinct VSGs deprived of shared conserved T cell epitopes [22], and (ii) there are invariable surface molecules that are needed by the parasite for nutrient binding and uptake, the parasite had to acquire additional defense mechanisms. This secondary level of protection is obtained by rapid lateral surface movement and endocytosis of antibody-complexed surface molecules. This not only allows the removal of VSG-bound antibodies, but also reduces the efficacy of complement-mediated killing after C3b surface deposition [19,23]. This is important as IgM-mediated complement cascade activation and CR3-mediated parasite phagocytosis by macrophages are considered crucial anti-trypanosome clearance strategies [24]. An additional defense against C3b surface binding is provided by the shedding of soluble (s)VSG molecules, resulting in complexing the opsonin C3b away from the parasite membrane [25]. As trypanosomes also cause a rapid reduction in C1, C1q and C3 serum concentrations, it is clear that these parasites have adopted a range of strategies to reduce the risk of IgM/C3b-mediated elimination [26]. Finally, trypanosomes also severely compromise the host’s antibody production capacity by exerting a detrimental effect on the B cell compartment itself [15,27,28,29]. The cytotoxic effect of NK cells and contact-dependent interactions between B cells and trypanosomes, which induce the apoptosis of transitional B cells after caspase activation in conjunction with CD95 surface upregulation, both suggest impairment of the immune system. Caspase-3-dependent apoptosis of marginal zone B cells coinciding with the downregulation of the anti-apoptotic marker Bcl-2 as well as TNFRSF13C, encoding the surface-expressed B cell maturation and survival receptor BAFF-R, delivers an additional damaging effect to the antibody production capacity [15,27,30,31,32].

As trypanosomes have a severe detrimental impact on the mammalian adaptive immune response, there has been no progress made towards a field applicable vaccine [17]. The problem of trypanosomosis is exacerbated by the fact that field data have shown infections result in failures of unrelated veterinary vaccines, such as those for classic swine fever [33], foot and mouth disease [34], *Pasturella multocida* [35], and *P. haemolytica* [36], and cause overall susceptibility to secondary infections [37]. While this pathology aspect of trypanosomosis in HAT is not well studied, a study addressing the measles vaccine interference in HAT patients showed a significant infection-associated reduction in vaccine-induced antibody titers, although the functional consequence of the latter was not addressed [38]. Interestingly, the antibody titers did not recover after therapeutic anti-HAT treatment. In a more experimental vaccine setting, the first data that showed the detrimental impact of trypanosomosis on heterologous vaccine-induced memory in mouse models involved the *Diphtheria Tetanus Pertussis* ‘acellular’ (DTPa) vaccine [30]. This human combination vaccine is routinely used to raise protection against diphtheria, tetanus, and pertussis. It consists of diphtheria toxoid, tetanus toxoid and three purified antigens of *Bordetella pertussis*, i.e., pertussis toxoid (PT), pertussis filamentous haemagglutinin (FHA) and pertactin (PRN), adsorbed onto aluminum salts. Using an antigen cocktail, rather than a whole-cell approach, reduces the inflammatory side effects. While this vaccine has been shown to be very effective in safeguarding mice against a nasal *B. pertussis* challenge, protection is totally lost in mice that are suffering from an ongoing experimental *T. b. brucei* infection [30].

Given the vast size of the wildlife trypanosome reservoir, only a vaccine-based approach will be able to lift the global burden of this disease. Indeed, while the active case detection and targeted treatment of human African trypanosomosis (sleeping sickness) has been successful in recent years [39], zoonotic trypanosomosis control requires a sustainable vaccination approach, as eradication of the reservoir is considered to be impossible. The same counts for the protection of livestock animals, in which prolonged drug treatment causes undesirable side effects. These include the risk of drug resistance buildup in the parasite population, and the toxicity problems related to the presence of drug residues in animal products such as milk and meat, especially when extremely dangerous treatment regiments are used [40]. Hence, understanding the mechanism of trypanosomosis-induced vaccine failure, as a result of the permanent destruction of the immune memory compartment, needs to be thoroughly addressed. Here, this pathology aspect was tackled by readdressing the DTPa/*T. b. brucei* heterologous vaccine setting, showing that curative anti-parasite treatment does allow full naïve immune recovery in mice, but fails to restore the pre-existing vaccine-induced protective memory responses. In previous research, we have shown that active *T. b. brucei* infections suppress the protection of a commercial DTPa vaccine against *B. pertussis* [30]. This observation was in line with other reports showing the detrimental impact of ongoing trypanosome infections on commercial veterinary vaccines [33,34,35,36]. However, none of these studies addressed whether the trypanosomosis-associated damage to the immune system was permanent, or merely the result of immunosuppression caused by the ongoing parasite infection.

## 2. Materials and Methods

### 2.1. Mice and Parasites Infections

Female 7- to 9-week-old BALB/c were purchased from Koatech, South Korea. The pleomorphic AnTat 1.1E (EATRO 1125 stock) *T. brucei brucei* was used as previously described, infecting mice by intraperitoneal (i.p.) injection of 5000 parasites/mouse [41]. Every 2 to 3 days, the number of parasites present in the blood was counted using a hemocytometer and light microscope and a 2.5 μL blood sample collected from the tail vein diluted 1/200 in DPBS (Invivogen, San Diego, CA, USA). Parasitemia was recorded for a total period of 15 days. All experimental mouse procedures were approved by the GUGC Institutional Animal Care and Use Committee (IACUC) (file # 2018-012).

### 2.2. DTPa Vaccine Procedure and In Vivo B. pertussis Challenge

Mice were vaccinated according to the previously published protocol [30]. In short, vaccination was done using 1/4 of a human dose of the DTPa vaccine (GlaxoSmithKline, Rixensart, Belgium) administered subcutaneously (s.c.) in the scruff of the neck. After 21 days, mice received a booster injection (s.c.) with the same amount of vaccine. After a further 14 days, mice were infected i.p. with 5,000 *T. b. brucei* parasites/mouse. Two weeks post-infection, mice received a curative 40 mg/kg dose of diminazene diaceturate (Veriben^®^ CEVA, Libourne, France) and were allowed to recover for 6 weeks, prior to intranasal challenge with a dose of 5 × 10^6^ *B. pertussis* bacteria/mouse (ATCC 9797 reference strain) in 10 μL DPBS (Invivogen, San Diego, CA, USA). Control mice received the intranasal *B. pertussis* challenge in the absence of a trypanosome challenge, or in the absence of vaccination and parasite infection. Lung bacterial load clearance was monitored after 18 h, and 3, 6, 9 days post-challenge, the latter being the humane endpoint for mice that failed to control infection. Mice were sacrificed and whole lungs were isolated and homogenized in 5 mL DPBS (Invivogen, San Diego, CA, USA). Serial 10-fold dilutions were prepared and aliquots of 200 μL were plated onto the Bordet-Gengou agar plates (Merck, Darmstadt, Germany, Cat. No. B4551). The number of colony forming units (CFUs) was counted after 72 h of incubation at 37 °C. All experimental mouse procedures were approved by the GUGC Institutional Animal Care and Use Committee (IACUC) (file # 2018-012).

### 2.3. Quantification of Cytokines by ELISA

Cytokine quantification was done by enzyme-linked immunosorbent assay (ELISA) using the mouse IFNγ and TNF MAX^TM^ Deluxe sets (BioLegend, San Diego, CA, USA, Cat. No. 430804 and 430904). In short, heparinized plasma was collected at several timepoints throughout the experiment and stored in aliquots at −20 °C. After thawing, plasma was diluted 1/2 in the assay diluent provided by the manufacturer and processed according to the kit’s protocol. 

### 2.4. Quantification of Anti-Pertussis and Anti-VSG Antibody Titers by ELISA

*B. pertussis* was cultured at 37 °C in Stainer–Scholte broth (SS) medium, seeding bacteria at approximately OD = 0.2 and harvesting cells 30 h later at OD = 1. Whole cells were pelleted by centrifugation for 10 min at 5000× *g* and resuspended in ice-cold TE buffer. Cells were centrifuged for 10 min at 5000× *g* and resuspended in 1 mL 0.04 M lysozyme/TE buffer followed by incubation for 35 min at 37 °C. Samples were sonicated using 5 cycles of 10-s burst/30-s cooling on ice, diluted in 4 mL PBS pH 7.2 and centrifuged for 30 min at 16,000× *g* , at 4 °C. Protein concentration in the supernatant was determined by Bradford assay (Thermo Scientific, Waltham, MA, USA, kit Cat. No. 23200) and stored at −20 °C. Lysate was coated in 96-well half-area clear flat bottom polystyrene high-bind microplate (Corning Inc., Corning, NY, USA) at 4 °C overnight, coating 0.1 µg/50 µL/well, using a 0.05 M bicarbonate 9.6 pH coating buffer (3.7 g sodium bicarbonate (NaHCO_3_)/0.64 g sodium carbonate (Na_2_CO_3_)/1 L H_2_O). Heparinized plasma was collected from infected mice at several timepoints throughout the experiment and stored in aliquots at −20 °C. Plasma IgG1 and IgG2a titers were determined using horseradish peroxidase-labeled specific secondary antibodies (Southern Biotech, Birmingham AL, USA, kit Cat. No. 5300-05). Anti-VSG titers were measured as described before [42], using purified AnTat 1.1 VSG as coating (0.1 µg/50 µL/well, using a 0.05 M bicarbonate 9.6 pH coating buffer) and 96-well half-area clear flat bottom polystyrene high-bind microplate (Corning Inc., Corning, NY, USA). Total plasma Ig titers, IgM titers and IgG2a titers, were determined using horseradish peroxidase-labeled specific secondary antibodies (Southern Biotech, Birmingham AL, USA, kit Cat. No. 5300-05). TMB (Sigma, St. Louis, MO, USA Cat. No. T0440) substrate conversion was measured at 450 nm (with 570 nm as background wavelength) using a Multiskan *PC* ELISA reader (Thermo Scientific, Waltham, MA, USA). For all ELISA assays, plasma was diluted 1/200 as the start concentration and subsequently diluted further as a 1:2 serial dilution up to 1/51.200, using DPBS (Invivogen, San Diego, CA, USA). 

### 2.5. Cell Preparation and Flow Cytometry Analysis

Spleen cells were isolated at different timepoints. Single-cell suspensions were prepared by homogenizing spleens in 6 mL of DMEM (Capricorn Scientific, Ebsdorfergrund, Hessen, Germany) supplemented with 10% FBS (Atlas Biologicals, Fort Collins, CO, USA) and 1% penicillin/streptomycin using gentleMACS™ Dissociator (Miltenyi Biotec, Bergisch Gladbach, Germany). After passing the homogenate through a 70-µm cell strainer (SPL Life Sciences, Gyeonggi-do, South Korea), cells were centrifuged at 314× *g* for 7 min at 4 °C, followed by re-suspension and incubation in RBC lysis buffer (BioLegend, San Diego, CA, USA) at 4 °C for 5 min. After washing (314× *g* 7 min at 4°C), cells were kept on ice in FACSFlow sheath fluid (BD Biosciences, San Jose, CA USA) containing 0.05% FBS (Atlas Biologicals, USA) and Fc block (CD16/CD32 Fcγ III/II, BioLegend, San Diego, CA, USA) (1/1000 dilution) for 30 min in the dark at 4 °C. Subsequently, 10^5^ cells per sample were incubated for 30 min in the dark at 4 °C, with antibody cocktails specific for different splenocytes populations, followed by flow cytometry analysis using a BD Accuri™ C6 Plus flow cytometer (BD Biosciences, San Jose, CA, USA). The percentage of each population was determined by dividing the number of events within a series of marker negative or positive gates, by the total number of events within live gate. The gating strategies were used in a previously published study [43].

### 2.6. Flow Cytometry Detection Reagents

The following antibodies (BioLegend, San Diego, CA, USA) were added to 100 µL aliquots of 10^5^ Fc-blocked splenocytes prepared as described above to make a final 1/600 dilution: anti-CD1d-PE(clone 1B1), anti-B220-FITC (clone RA3-6B2), anti-CD93-APC (clone AA4.1), anti-CD138-PE-Cy7 (clone 281-2), anti-GL7-PE (clone GL7), anti-Ly6G-Alexa488 (clone 1A8), anti-Ly6C-PE (clone HK 1.4), anti-CD4-FITC (clone GK 1.5), anti-CD8-PE (clone 53-6.7), anti-NK1.1-APC(clone PK 136), anti-TCRβ-PE-Cy7 (clone H57-597).

### 2.7. Statistical Analysis

GraphPad Prism v.8.3 (GraphPad Software Inc., San Diego, CA, USA) was used for final data presentation and statistical result analysis. Unless otherwise stated, data were compared with naïve using Student’s *t*-test. Means are given as ± standard deviation (SD). 

## 3. Results

### 3.1. T. b. brucei Destroys DTPa Vaccine-Induced Protection against Bordetella Pertussis

**Figure 1:**
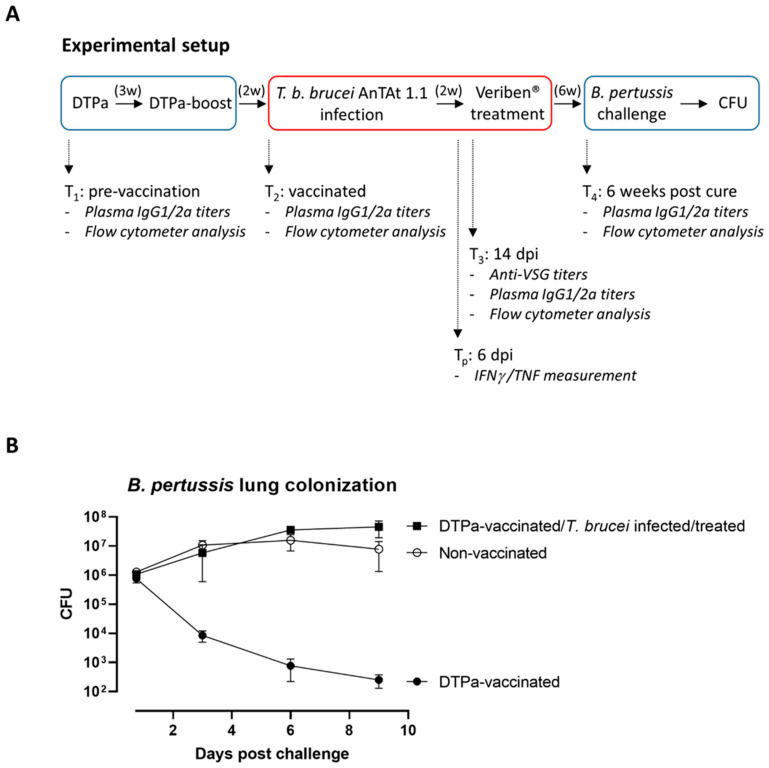
(**A**) Experimental layout. Mice were vaccinated and boosted with DTPa with a three-week interval, followed by intraperitoneal challenge with 5000 *T. b. brucei* AnTat 1.1 parasites. Two weeks into the infection all mice were treated with diminazene diaceturate (40 mg/kg). After a 6-week recovery period, mice were exposed to an intranasal challenge with 5 × 10^6^ *B. pertussis* bacteria (ATCC 9797 reference strain) in 10 μL DPBS. Subsequently, lung bacterial load clearance was monitored by CFU determination. Anti-pertussis plasma antibody titer determination (IgG1 and IgG2a) as well as flow cytometry analysis of the spleen was executed at T_1_, T_2,_ T_3_ and T_4_. Plasma ELISAs for IFNγ and TNF were performed at the peak of the *T. b. brucei* infection T_p_ (peak timepoint = 6 dpi). Control groups included naïve mice, non-vaccinated *T. b. brucei*-infected mice and DTPa-vaccinated mice that were not challenged with trypanosomes. (**B**) Experimental infection with *T. b. brucei* leads to the permanent abrogation of DTPa vaccine protection against *B. pertussis*. Bordet-Gengou agar plate lung homogenate CFUs were determined after 18 h as well as after 3, 6 and 9 days of infection. CFUs were measured after 72 h incubation and are represented as the mean ± SD of three individual mice per timepoint.

Mice were vaccinated and boosted with the DTPa vaccine over a period of 5 weeks prior to exposure to a *T. b. brucei* AnTat 1.1 infection. Two weeks into the trypanosome infection, the mice were cured using a standard treatment of diminazene diaceturate (40 mg/kg) and allowed to recover for six weeks. Subsequently, the mice received an intranasal challenge with 5 × 10^6^ *B. pertussis* bacteria, after which lung CFUs were estimated (Figure 1A). Under control conditions, the DTPa vaccine does offer adequate protection against *B. pertussis*, with lung CFUs rapidly declining over a 9-day monitoring period (Figure 1B). In contrast, DTPa-vaccinated mice that had been exposed to the *T. b. brucei* infection eight weeks earlier, subsequently cured and allowed to recover for a 6-week period, completely failed to mount a protective vaccine recall response.

### 3.2. T. b. brucei Infection Alters the IgG1/IgG2a Ratio of Anti-Pertussis Antibody Titers in DTPa-Vaccinated Mice

**Figure 2:**
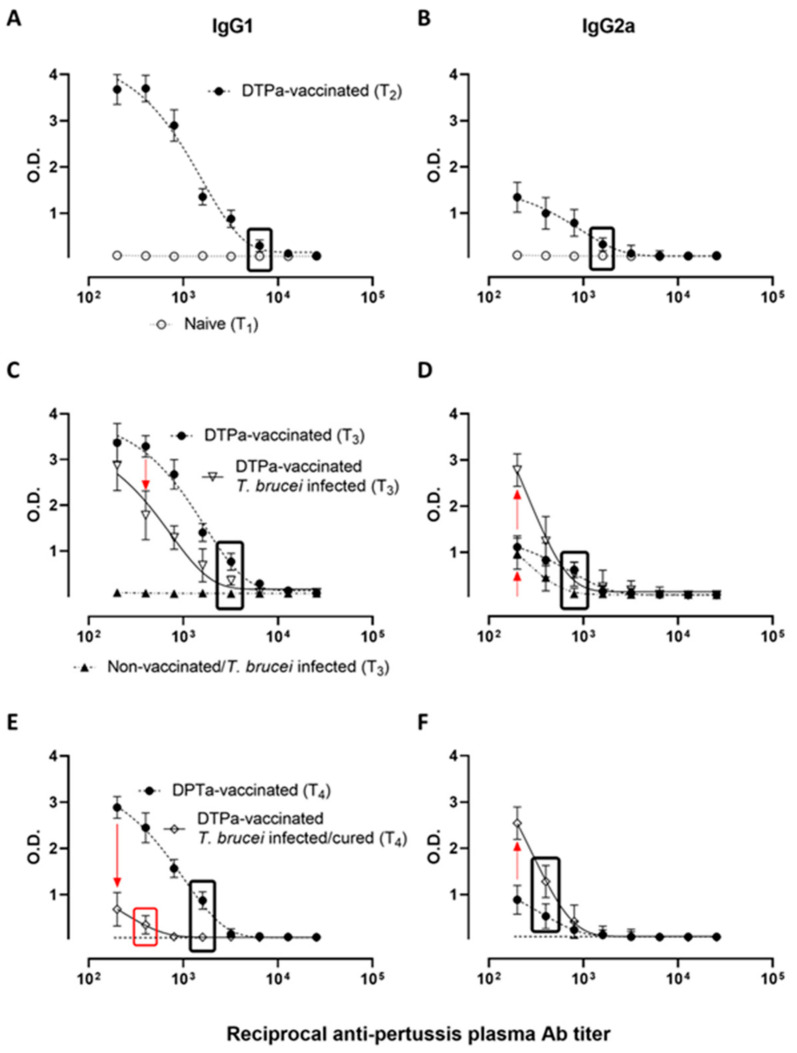
DTPa-induced IgG1 (left panels) and IgG2a (right panels) titers are strongly affected by *T. b. brucei* infection. (**A**,**B**) Plasma samples were collected prior to the start of the DTPa vaccination (T_1_), and two weeks after the vaccine boost (T_2_). (**C**,**D**) Plasma samples were collected at experimental timepoint T_3_ from both DTPa-vaccinated and non-vaccinated *T. b. brucei* AnTat 1.1 challenged mice (14 dpi) as well as control DTPa-vaccinated mice that were not infected by *T. b. brucei*. (**E**,**F**) Plasma samples were collected at experimental timepoint T_4_ from six-week cured DTPa-vaccinated mice that had recovered from their *T. b. brucei* infection. Control samples were obtained here from DTPa-vaccinated mice that had not been challenged with *T. b. brucei*. Plasma dilution series (1:2) were made to determine anti-*B. pertussis* antibody endpoint titers for all experimental groups indicated as black boxed data points. This value represents the last serial dilution resulting in an OD that is significantly different from the OD value obtained at the same dilution in naïve mice (represented in d, e and f as a dashed line). The red boxed value in (**E**) indicates the significant *T. b. brucei*-induced reduction in IgG1 titers at T_4_. The red arrows indicate the *T. b. brucei*-induced trends in reduction (**C**,**E**) or increase (**D**,**F**) in anti-pertussis antibody ELISA OD readings. Values are represented as the mean ± SD of five individual mice per timepoint.

The protective effect of DTPa against *B. pertussis* has been associated with the strong IgG1-inducing potential of the vaccine, mostly reported by the high IgG1/IgG2a ratio of DTPa-induced antibodies [44,45]. Here, we recorded plasma anti-pertussis antibodies in vaccinated mice, compared to vaccinated mice infected with *T. b. brucei* AnTat 1.1. Following the vaccination and boost (T_2_ compared to T_1_), all of the mice had significant IgG1 titers against the soluble fraction of the total bacterial *B. pertussis* lysate, with the endpoint titers reaching 1/6400 (Figure 2A). The vaccination-induced IgG2a titers were four times lower compared to the IgG1 titers (Figure 2B). Two weeks into the *T. b. brucei* infection (T_3_), the DTPa-induced IgG1 titers showed a downwards trend, showing a two-fold reduction compared to the pre-trypanosome challenge timepoint. The *T. b. brucei* infection itself did not induce any cross-reactive anti-pertussis IgG1 antibodies (Figure 2C). Also, for the anti-pertussis IgG2a antibody levels, a two-fold reduction was observed when comparing the endpoint titers between the pre-infection (T_2_) and 14 dpi data, with the titers reaching only 1/800 at T_3_. However, in contrast to the IgG1 results, dilutions 1/200 and 1/400 show a clear trypanosomosis-associated induction of IgG2a antibodies. In this case, also the non-vaccinated infected mice (T_3_, 14 dpi) showed significant IgG2a antibody levels against the soluble fraction of the total bacterial *B. pertussis* lysate, reaching the same 1/800 endpoint titers as the DTPa-vaccinated infected mice. These data indicate that the IgG2a titers measured in this assay resulted from polyclonal activation of the adaptive immune system, caused by infection-induced inflammatory pathology (Figure 2D). Six weeks post-treatment (T_4_) the antibody titers gradually declined, once again two-fold, in the DTPa-vaccinated control mice due to natural antibody clearance. In contrast, the mice that had been exposed to the *T. b. brucei* infection, and subsequently cured, showed a much greater reduction in IgG1 levels both in terms of absolute ELISA OD readings as well as endpoint titers, with the latter only reaching 1/400 (Figure 2E). In contrast, the cross-reactive *T. b. brucei*-induced IgG2a antibody levels remained high in the ELISA OD readings at low plasma dilutions (1/200 and 1/400), while the endpoint titers dropped to the same levels as those for IgG1, being 1/400 (Figure 2F).

### 3.3. T. b. brucei Infection Gives Rise to the Rapid Destruction of the Host Spleen B Cell Compartment, While Anti-Trypanosome Treatment Results in a Full Cellular Spleen Recovery

**Figure 3:**
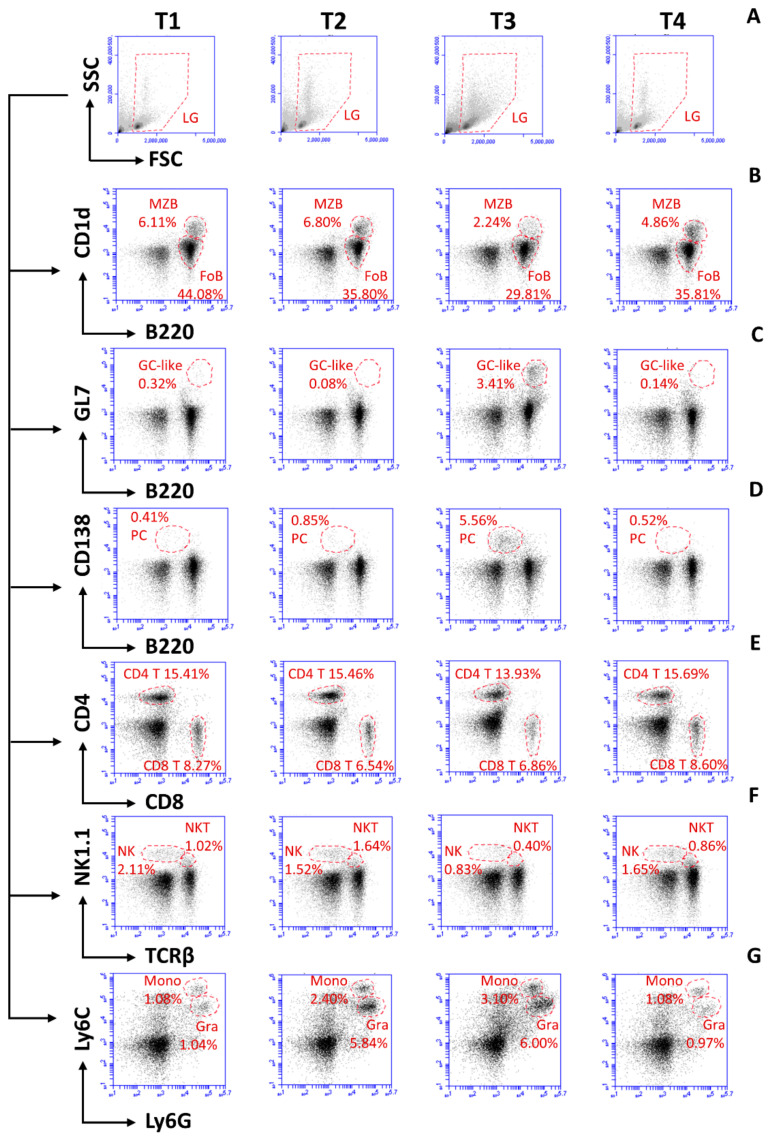
Early-stage *T. b. brucei* infection has a detrimental impact on the spleen composition in DTPa-vaccinated mice. Flow cytometry analysis of spleens of naïve mice (T1), DTPa-vaccinated mice prior to parasite challenge (T2), DTPa-vaccinated *T. b. brucei* AnTat 1.1-infected mice (T3), and DTPa-vaccinated *T. b. brucei* AnTat 1.1 challenged and cured mice (T4). Analysis was performed on (**A**) live-gated cells, identifying: (**B**) CD1d^+^ marginal zone and CD1d^-^ follicular B220^+^ B cells, (**C**) GL7^+^B220^+^ germinal center-like B cells (**D**) CD138^+^B220^Int^ plasma B cells, (**E**) CD4^+^ and CD8^+^ T cells, (**F**) NK1.1^+^ and NK1.1^+^TcRβ^+^ cells, and (**G**) Ly6C^Int^/Ly6G^High^ granulocyte and Ly6C^High^/Ly6G+ monocytes. One representative plot from a biological triplicate experiment is shown for each population.

**Figure 4:**
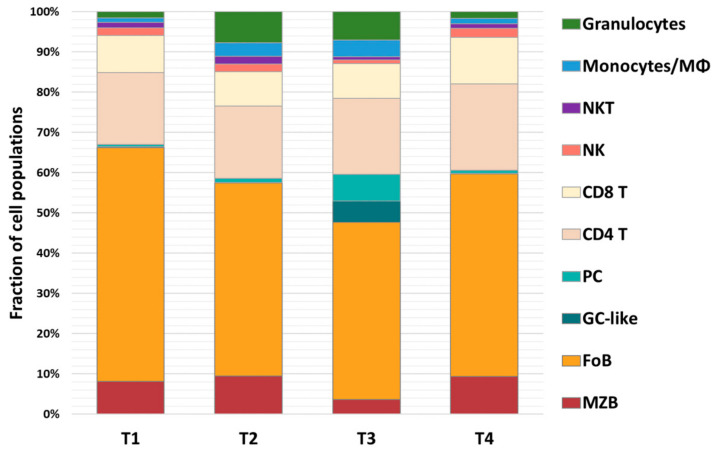
Early-stage *T. b. brucei* infection affects mainly the spleen B cell (reduction) and granulocyte (increase) numbers in DTPa-vaccinated mice. Numerical data, expressed as % value of the total spleen is plotted for marginal zone (MZB), follicular (FoB), germinal center-like (GC-like), and plasma B cells (PC), CD4+ and CD8+ T cells, NK1.1+ and NK1.1+TcRβ+ (NKT) spleen cells, and the monocyte/macrophage (Mφ) compartment as well as the spleen granulocyte population. Analysis was done for spleens of naïve mice (T1), DTPa-vaccinated mice prior to parasite challenge (T2), DTPa-vaccinated *T. b. brucei* AnTat 1.1-infected mice (T3), and DTPa-vaccinated *T. b. brucei* AnTat 1.1 challenged and cured mice (T4).

*T. b. brucei* AnTat 1.1 infections trigger the rapid destruction of the spleen architecture and alteration of the spleen cell populations [30]. Hence, flow cytometry analysis was implemented to track this infection-associated pathology, as well as immune recovery after anti-trypanosome drug treatment. Measurements were taken using naïve mice (T_1_), DTPa-vaccinated mice (T_2_), DTPa-vaccinated/trypanosome-infected mice (T_3_) and vaccinated cured mice, just prior to the *B. pertussis* challenge (T_4_). The results are presented as flow cytometry measurements (Figure 3) as well as total spleen cell number counts (Figure 4). While the spleens of the vaccinated mice show a similar cellular composition to those of the naïve mice for most of the cell compartments (Figure 3 T_1_, compared to Figure 3 T_2_), a marked increase in Ly6G^High^Ly6C^Int^ granulocytes occurs after the vaccination (Figure 3 T_1_f compared to Figure 3 T_2_f). This observation is confirmed when the absolute spleen cell numbers are calculated (Figure 4). Two weeks into the infection (T_3_), trypanosomosis-associated destruction of the B220^+^CD1d^+^ marginal zone B cell compartment and the B220^+^CD1d^Low^ follicular B cell compartment is prominent (Figure 3 T_3_a). This coincided with an increase in CD138^+^ Plasma B cells (Figure 3 T_3_b) and GL7^+^ germinal center-like B cells (Figure 3 T_3_c). The CD4^+^ and CD8^+^ populations remain almost unchanged (Figure 3 T_3_d), while both the NK1.1^+^ and NK1.1^+^TcRβ^+^ T cell populations decline (Figure 3 T_3_e). During the infection, the Ly6C^Int^/Ly6G^High^ granulocyte cell count increases, accompanied by an influx of Ly6C^High^/Ly6G+ monocytes and macrophages (Figure 3 T_3_f). Six weeks post-treatment, all of the cell counts regain a status that is roughly comparable to the naïve mice (Figure 3 T_4_). However, as shown in Figure 1B, this did not result in a restoration of the previously acquired DTPa-induced vaccine protection against *B. pertussis*.

### 3.4. DTPa Vaccination Results in the Temporary Improvement of Trypanosomosis Control Coinciding with an Increased Anti-Parasite IFNγ Response

**Figure 5:**
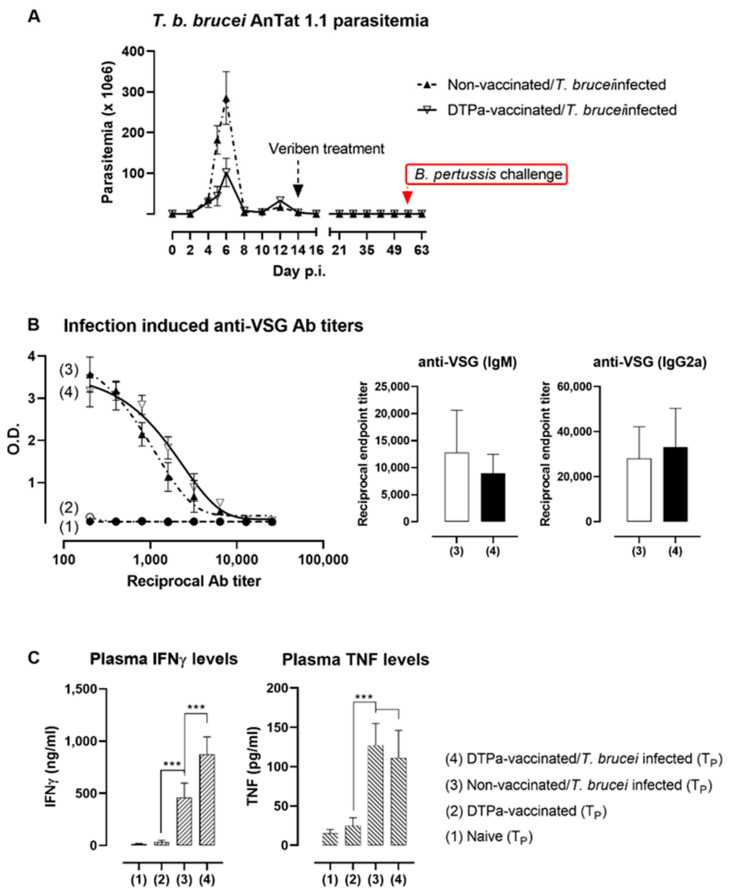
DTPa vaccination results in the temporary improvement of *T. b. brucei* control. (**A**) Parasitemia in DTPa-vaccinated and non-vaccinated mice was recorded at 2- to 3-day intervals during the first two weeks of infection, and one-week intervals after curative drug treatment (up to 63 dpi). All mice were infected through i.p. injection of 5000 *T. b. brucei* AnTat 1.1 trypanosomes. (**B**) Anti-trypanosome antibody titers were measured in a VSG-specific ELISA format with samples derived from naïve mice (1), DTPa-vaccinated mice (2), non-vaccinated *T. b. brucei*-infected mice (3) and DTPa-vaccinated *T. b. brucei*-infected mice (4). All samples were analyzed at timepoint T_3_, using 1:2 plasma dilutions. Anti-VSG IgM antibody titers and IgG2a titers were measured and used for endpoint titer calculation for non-vaccinated *T. b. brucei*-infected mice (3) and DTPa-vaccinated *T. b. brucei*-infected mice (4). (**C**) Plasma INFγ and TNF concentrations were determined by ELISA in naïve mice (2) and DTPa-vaccinated mice (2) as well as in both non-vaccinated and DTPa-vaccinated *T. b. brucei* AnTat 1.1 challenge mice at 6 dpi (T_p_). Values are represented as the mean ± SD of five individual mice per timepoint. ∗∗∗ indicates a significance level of *p* ≤ 0.001.

During the execution of the experimental setup outlined above, the parasitemia development of *T. b. brucei* AnTat 1.1, as well as the subsequent treatment success, were monitored. This was done in both DTPa-vaccinated and non-vaccinated mice. Interestingly, DTPa exposure itself appeared to have a parasite suppressive effect, as the peak parasitemia in the vaccinated mice were significantly reduced (Figure 5A). Two weeks into the infection, all of the mice were drug-cured, and were found to be parasite-free for the remaining seven weeks of the experiment. As there was no reason to assume that the DTPa-induced antibody responses had any parasitemia altering effects, the total anti-trypanosome (VSG) antibody titers were measured in the DTPa-vaccinated and non-vaccinated experimental groups, both before *T. b. brucei* infection and two weeks into the infection. The data confirms that the DTPa vaccination itself did not induce any cross-reactive anti-VSG antibodies (Figure 5B). The measurement of specific IgM and IgG2a titers, the two main antibodies induced against *T. b. brucei* in BALB/c mice, confirmed this finding. Hence, an explanation for the improved parasitemia control in the DTPa-vaccinated mice had to coincide with another vaccination-associated immune parameter. Interestingly, when the plasma IFNγ levels were measured in all of the experimental groups at the peak stage of the infection (T_p_), both non-vaccinated mice as well as DTPa-vaccinated mice exhibited increased circulating cytokine levels, with the latter being significantly higher (Figure 5C). In contrast, the TNF plasma levels were not affected by pre-exposure to the DTPa vaccine and were infection driven, being identical in both the non-vaccinated and DTPa-vaccinated mice at the timepoint of peak parasitemia (T_p_).

## 4. Discussion

Salivarian trypanosomes cause diseases in humans, livestock, and game animals throughout most of the developing world. Human trypanosomosis is mostly confined to Africa, where it occurs as the following two distinct diseases: the West African and central African sleeping sickness, caused by *Trypanosome brucei gambiense* and the East African *T. b. rhodesiense* HAT. While there has long been a call for the development of a vaccine strategy for HAT, recent successes in the control of *T. b. gambiense* HAT have shown that persistent surveillance of the human population at risk, combined with dedicated drug treatment and vector control, can bring down the disease burden even without access to a vaccine intervention strategy. In fact, it is expected that by 2030, HAT should no longer be considered as a significant human threat [46]. With *T. b. gambiense* historically being responsible for over 95% of all HAT cases, this has to be considered as a great ‘global-effort’ success, as it involved many players in a consorted south–north strategy [47]. However, *T. b. gambiense* is an anthroponotic parasite, placing it in a rather unique context in terms of disease control. The control of *T. b. rhodesiense* HAT, on the other hand, is a completely different issue. This is a zoonotic infection in which the parasite reservoir is not found in the human population, but in livestock and game animals that roam East Africa. In such a setting, infection surveillance and targeted treatment becomes a virtually impossible task, and vaccination of the commercial animal reservoir is the only long-lasting sustainable solution to prevent the intermediate step in disease transmission from a wildlife reservoir to the human population. Besides this, there are other reasons why anti-trypanosome vaccine development is still on the agenda of many health organizations. First, there are a number of trypanosomes (*T. congolense*, *T. vivax* and *T. evansi*) that cause considerable livestock losses without posing a direct risk to human health [6]. These parasites obviously are the cause of economic hardship, preventing the human development of mainly smallholder farmers. For *T. vivax* and *T. evansi*, this includes vast territories in South America and Asia, as these parasites have been able to move out of Africa through non-tsetse mechanical transmission [48]. 

The treatment of animal infections is often done through the herd approach, without proper diagnosis, and with drugs that can cause serious residue issues in products such as milk and meat. For example, ethidium bromide is currently still being used and advertised as an anti-trypanosome drug, and is freely available for online purchase as an ‘injectable solution’ for the treatment of animal trypanosomosis. Obviously, this results in the fact that anti-trypanosome treatment itself becomes a direct health hazard for humans. Together, these finding show that anti-trypanosome vaccination should still be considered as the ultimate goal in the fight against both animal and human trypanosomosis, particularly limiting zoonotic transmissions. Unfortunately, despite many promising laboratory reports, no single vaccine-based solution has found its way into field application so far [17].

Anti-trypanosome vaccination is hampered by a number of fundamental problems, of which some have only become clear in recent years. In order to understand the difficulties in vaccine development for trypanosomosis, one should take a holistic view at the biological niche occupied by this parasite, as follows: Salivarian trypanosomes have evolved to be extracellular free-living parasites that dwell throughout the blood and lymph, in plain sight of the adaptive immune system, the host’s antibodies and the complement system. This is where they thrive; this is not a hostile environment for these parasites. This is also a biological niche where trypanosomes encounter virtually no resource competition from other pathogens or microorganisms. For decades, it has been known that the antigenic variation of the variant surface glycoprotein (VSG) coat plays a key role here as it (i) allows regular escape from high-affinity binding antibodies, by surface-exposed epitope alteration [16], (ii) allows the rapid clearance of surface-bound antibodies through endocytosis [19], and (iii) prevents complement-mediated lysis, both through its physical barrier function and antigen-shedding capacity [25]. However, looking at the fundamental way the adaptive immune system works, it is clear that antigenic variation per se would not allow trypanosomes to ‘eternally’ outrun the mammalian defense system. Indeed, the main issue any trypanosome would face is the fact that VSG molecules do not just have B cell epitopes that trigger antibody production, but they also have T cell epitopes that are vital in the development of the T cell help needed for B cell activation, differentiation and affinity maturation. Many of these T cell epitopes are conserved [22], meaning that once T cell help has been generated by one early VSG variant, B cell maturation against newly arising variants can immediately take place, with the help of existing T helper cells. Secondly, while molecular biology approaches have shown that individual trypanosomes can harbor over a thousand different VSG genes and pseudogenes [49], it has never been proven that these genes all encode for antigenically distinct antigens that are not being recognized by cross-reacting antibodies. Together, this makes it likely that, while trypanosomes can use VSG antigenic variation to establish a successful early-stage infection, the system would not provide an efficient defense system for long-term survival inside a given mammalian host. In order to ensure the latter, trypanosomes had to adopt a second layer of defense, i.e., the induction of B cell dysfunction, rendering these cells inefficient in producing truly detrimental antibody responses. This pathology involves the disruption of regular B cell affinity maturation pathways, as well as the induction of low-affinity polyclonal antibody responses that result in an antibody ‘dilution’ response [50]. While most of the data documenting this has been obtained using experimental mouse infection models, multiple reports of *T. evansi*-induced immune suppression show that B cell dysfunction is a real pathology in animal trypanosomosis [37]. For HAT, this problem has long been known [51], but the situation is less well studied, with one thorough study describing the damaging effect of *T. b. gambiense* infections on the vaccine-induced anti-measles response [38], and one study describing the problem of HAT-induced non-specific polyclonal B cell activation hampering HIV diagnosis [52]. In an experimental setting, we have previously shown that the human DTPa vaccine loses efficacy during active *T. b. brucei* infection. This observation could be explained by the multiple levels of immune suppression that are induced during active trypanosomosis [53,54,55,56]. In contrast, the study here provides data showing that the problem at hand is the active destruction of the functional host immune memory compartment. This extends to vaccine-induced memory responses that do not recover after anti-trypanosome treatment. The nature of the damage has to be related to the vast destruction of both the bone marrow and peripheral B cell compartments that are induced during infection [15,27,30]. While anti-trypanosome treatment allows a full recovery of these compartments, it appears that the host B cell lymphopoiesis in this case is reset to a naïve condition, while the pre-existing memory that was eradicated by the trypanosome did not recover. This trypanosomosis-associated pathology makes perfect sense in the biology of VSG antigenic variation. Indeed, the destruction or dysfunction of immunological memory would allow the parasite to escape from the buildup of cross-reactive detrimental antibodies, and even with time the re-use of previously expressed VSGs or nearly identical VSGs. The fact that non-related immune memory (such as the one induced by the DTPa vaccine) is destroyed during the infection has to be considered as collateral damage. Importantly, the experimental vaccine setting used here does not determine whether the final immune dysfunction was due to B cell compartment or T cell compartment damage. Both have previously been shown to be important for immunity against *B. pertussis* [57,58], and both have been reported to be affected by trypanosomosis [43]. Interestingly, previous DTPa vaccine data has shown that protective responses coincided with increased IgG1 antibody titers. In line with these findings, our results show that the breakdown of DTPa-induced protection against *B. pertussis* coincided with a very significant drop in anti-*B. pertussis* IgG1 plasma endpoint titers, from 1/1600 down to 1/400, at the timepoint where the intranasal bacterial challenge was administered (T_4_ in this setup). The ablation of protection also coincided with the trypanosomosis-induced deregulation of the spleen B cell compartment, and a trypanosome-induced bias towards IgG2a antibody production. This Ig isotype switch coincides with the presence of high *T. b. brucei* infection-associated IFNγ levels, which further enhances the vaccine-associated IFNγ response [59,60]. However, IgG2a *pertussis*-binding antibodies that are triggered during the trypanosome infection appear to be of low affinity, as binding is rapidly lost with increasing plasma dilutions. Hence, we conclude that the observed IgG2a induction has the typical characteristics of a polyclonal B cell activation response caused by the parasite in the immunological environment of a pathological trypanosomosis-associated inflammatory type-1 IFNγ context [61,62], and is unable to confer any detrimental biological activity against *B. pertussis*. Finally, the trypanosomosis-associated IFNγ response is most likely also linked to the immune destructive effect observed at the level of the spleen B cell compartment [63], and could also explain the spleen macrophage/granulocyte expansion observed during trypanosomosis [43]. Previous data has shown that the source of the infection-associated IFNγ is complex, involving NK, NKT, CD8^+^ and subsequently CD4^+^ T cells, cell populations that all decrease in size during *T. b. brucei* infections, but increase their cytokine secretion [62]. Whether or not the relative contribution of these populations for IFNγ production changes in the combined DTPa/*T. b. brucei* challenge was not addressed here. Such analysis could be included in future studies. Of interest, however, is that the increased levels of IFNγ in the combined DTPa/*T. b. brucei* challenge coincided with an improved peak parasitemia control. This could be explained by an improved clearance of the parasites by IFNγ-stimulated macrophages [24], and the fact that IFNγ itself is known as a resistance factor in *T. b. brucei* control, involving innate TLR/MyD88 signaling [64,65,66]. In this context, also TNF has been described as a cytokine involved in the peak parasitemia control of *T. b. brucei* parasites [67,68,69]. However, here, we found no DTPa-driven alterations of the plasma TNF levels, suggesting that the observed improved peak control in the DTPa/*T. b. brucei-*challenged mice was unrelated to TNF-mediated parasitemia control. The improved parasitemia control did also not coincide with any measurable enhanced host antibody response. 

When considering the future of anti-trypanosome vaccination, one last immunological/infection hurdle that needs to be considered is the speed at which the mammalian immune system can trigger an immune memory recall response before infection-associated B cell destruction sets in. Our data, and those of others, have shown that trypanosomes initiate the destruction of the host B cell compartment within the first week of infection [27,28,29]. The destruction of T cell functionality further undermines humoral immune activity [69]. This has to be the result of evolutionary pressure, as the latter is a race between parasites that try to undermine the immune system and the immune system that tries to eliminate the parasites. With trypanosomes being very successful organisms that are capable of infecting virtually any mammal, it is clear who has gained the upper hand in this race. Hence, even if a vaccine was to be developed against a non-variable conserved surface-exposed trypanosome molecule, such as a nutrient receptor, it remains to be seen whether a protective recall response could be triggered fast enough to stop the emergence of a first peak of parasitemia after an infectious challenge. Indeed, in this case, a successful approach would necessitate the full activation of the antibody production capacity within hours of infection, a requirement that appears unrealistic given the nature of the B and T cell memory recall responses. However, in a setting of regular pathogen exposure in endemic trypanosome areas, the need for a memory recall response might be replaced by the presence of a continued memory maintenance response. As such, efforts to develop an anti-trypanosome vaccine for the prophylactic protection of livestock animals should be continued, as it will not only protect agriculture economies, but also reduce the risk for zoonotic transmission, which could result in the re-emergence of human trypanosomosis [69].

## Data Availability

There are no publicly archived datasets analyzed in this study.
